# Zebrafish TRIM25 Promotes Innate Immune Response to RGNNV Infection by Targeting 2CARD and RD Regions of RIG-I for K63-Linked Ubiquitination

**DOI:** 10.3389/fimmu.2019.02805

**Published:** 2019-12-03

**Authors:** Yilin Jin, Kuntong Jia, Wanwan Zhang, Yangxi Xiang, Peng Jia, Wei Liu, Meisheng Yi

**Affiliations:** ^1^School of Marine Sciences, Sun Yat-sen University, Guangzhou, China; ^2^Southern Marine Science and Engineering Guangdong Laboratory (Zhuhai), Zhuhai, China; ^3^Guangdong Provincial Key Laboratory of Marine Resources and Coastal Engineering, Guangzhou, China

**Keywords:** TRIM25, RIG-I, ubiquitination, zebrafish, red spotted grouper nervous necrosis virus

## Abstract

RIG-I-like receptors (RLRs) play important roles in response to virus infection by regulating host innate immune signaling pathways. Meanwhile, the RLR signaling pathway is also tightly regulated by host and virus to achieve the immune homeostasis between antiviral responses and virus survival. Here, we found that zebrafish TRIM25 (zbTRIM25) functioned as a positive regulator of RLR signaling pathway during red spotted grouper nervous necrosis virus (RGNNV) infection. Post-RGNNV infection, zbTRIM25 expression was obviously inhibited and ectopic expression of zbTRIM25 led to enhanced expression of RLR signaling pathway-related genes. Overexpression and knockdown analysis revealed that zbTRIM25 promoted zebrafish RIG-I (zbRIG-I)-mediated IFN signaling and inhibited RGNNV replication. Mechanistically, zbTRIM25 bound to zbRIG-I; in particular, the SPRY domain of zbTRIM25 interacted with the tandem caspase activation and recruitment domains (2CARD) and repressor domain (RD) regions of zbRIG-I. zbTRIM25 promoted the K63 polyubiquitination of 2CARD and RD regions of zbRIG-I. Furthermore, zbTRIM25-mediated zbRIG-I activation of IFN production was enhanced by K63-linked ubiquitin, indicating that zbTRIM25-mediated zbRIG-I polyubiquitination was essential for RIG-I-triggered IFN induction. In conclusion, these findings reveal a novel mechanism that zbTRIM25 positively regulates the innate immune response by targeting and promoting the K63-linked polyubiquitination of zbRIG-I.

## Introduction

The innate immune system recognizes pathogen-associated molecular patterns (PAMPs) by pattern recognition receptors (PRRs) as against microbial pathogen invasion (1). Retinoic acid inducible gene-I (RIG-I)-like receptors (RLRs), as intracellular PRRs, composed of RIG-I, MDA5, and LGP2, recognize non-self signatures of viral RNAs in the cytosol of cells. After activated by viral RNA, RIG-I and MDA5 recruited the downstream adaptor molecule, MAVS, to their N-terminal caspase-recruitment domains (CARDs). Then, tumor necrosis factor receptor-associated factors (TRAF) and TANK-binding kinase 1/Iκ-B kinase ε interacted with MAVS, which in turn leads to the phosphorylation and cytoplasm-to-nucleus translocation of interferon (IFN) regulatory factor 3 (IRF3), and the activation of type I IFN. Subsequent IFNs activated a variety of IFN-stimulated genes (ISGs) to limit the virus replication (2).

Nervous necrosis virus (NNV) is a non-enveloped, single-stranded RNA virus belonging to the family *Nodaviridae*. Increasing evidence has shown that NNV can infect more than 120 fish species and causes mass mortalities of infected fish worldwide (3). It has been revealed that RLRs respond *in vivo* or *in vitro* to the stimulation of NNV and possess capacities in the induction of IFNs and ISGs in a variety of fish species. For example, in ZF4 cells, expression of RLRs was significantly enhanced post-NNV infection and RIG-I knockdown significantly restrained group II type I IFN activation (4). Our previous studies also suggested that RLR signaling pathway was activated during red spotted grouper nervous necrosis virus (RGNNV) infection in sea perch and its key components possessed anti-RGNNV activities (5, 6). However, regulation mechanisms of RLR signaling pathway during RGNNV infection is still unclear. RLR-mediated antiviral signaling pathway is tightly regulated at multiple steps in the signaling cascade. Several studies demonstrated that post-translational modifications, including ubiquitination, ISGylation, and phosphorylation, were important mechanisms that regulated the RLR signaling pathway, of which ubiquitination was a key regulatory mechanism for RLR pathway (2). For instance, RNF122 negatively regulated RLR signaling pathway by targeting RIG-I (7). MDA5 and MAVS were targeted for K48-linked ubiquitination by TRIM13 and RNF5, respectively, which induced MDA5 and MAVS degradation and RLRs signal termination (8, 9). TRIM25 E3 ubiquitin ligase induced the K63-linked ubiquitination of RIG-I, which activated RLR signaling pathway to elicit host antiviral innate immunity (10).

TRIM25, an IFN-inducible E3 ligase, is associated with all kinds of cellular processes, such as the immune response, cancer, and so on (11). It is becoming evident that TRIM25 has a dual role in RIG-I regulation, since TRIM25 not only induces K63-linked ubiquitination of RIG-I to positive regulate RLR signaling activation but also negatively regulates RIG-I activation through inhibiting HLA-F adjacent transcription 10 degradation, a negative regulator of RIG-I-mediated inflammatory response (12).

Multiple fish TRIM25 homologs have been reported, including *Rhodeus uyekii* (13), *Epinephelus coioides* (14), and *Larimichthys crocea* (15). Increasing evidence showed that fish TRIM25 was involved in antiviral immunity and played a pivotal role in RLR antiviral signaling pathway (14). However, the mechanism by which fish TRIM25 regulates RLR signaling pathway has not been explored. In the present study, zebrafish TRIM25 (zbTRIM25) was involved in RGNNV infection and was identified as a positive mediator of RLR signaling pathway by binding to and ubiquitinating the caspase activation and recruitment domain (2CARD) and repressor domain (RD) regions of RIG-I, which is different with the findings in mammals. Our findings reveal a novel mechanism of TRIM25 to activate RLR signaling pathway and will help to develop new treatments for viral nervous necrosis disease.

## Materials and Methods

### Ethics Statement

All procedures with zebrafish were approved by the Ethics Committee of Sun Yat-Sen University and the methods were carried out following the approved guidelines.

### Fish Strains, Cell Lines, Virus, and Reagents

Zebrafish wild-type AB line was purchased from China Zebrafish Resource Center. Fish were raised with 10 h darkness and 14 h light at 28°C and were fed with commercial pellets twice a day. All embryos were obtained by natural spawning and staged as previously reported (16).

ZBE3 cells derived from zebrafish embryos were cultured at 28°C as previously described (17). HEK 293T cells were cultured in DMEM (Invitrogen) enriched with 10% FBS (Invitrogen) at 37°C under a humidified atmosphere of air containing 5% CO_2_.

RGNNV was propagated in ZBE3 cells and stored at −80°C until use.

Anti-Flag (M20008), anti-Myc (M20002), anti-His (M20001L), and anti-HA antibodies (M20013) were purchased from Abmart. Anti-α-tubulin (ab15246) and anti-GFP antibodies (G1544) were purchased from Abcam and Sigma, respectively. Goat anti-rabbit IgG-HRP, goat anti-mouse IgG-HRP, Alexa Fluor 488-labeled goat anti-mouse IgG, and Alexa Fluor 555-labeled goat anti-rabbit IgG secondary antibodies were purchased from Invitrogen.

### Viral Challenge

For *in vitro* infection, ZBE3 cells were challenged with RGNNV [multiplicity of infection (MOI) = 1] for 6, 12, and 24 h, respectively. Subsequently, RNA from cells was extracted to detect the expression of *zbTRIM25* mRNA by quantitative real-time PCR (qRT-PCR).

For *in vivo* infection, RGNNVs (10^8^ TCID_50_/ml) were injected into the egg yolk of 50 embryos at the single-cell stage in the experimental group. In the mock group, 50 embryos were injected with DMEM. A total of 1 nl of solution was microinjected into each embryo using a microinjector. RNA from zebrafish embryo was extracted to detect the expression of *zbTRIM25* mRNA by qRT-PCR at 24 h post-injection.

### Knockdown of zbTRIM25 by siRNA

zbTRIM25 siRNA (5′-GAATCCAGTTGAAGAGAAA-3′) and control siRNA were synthesized by Ribobio Company (Guangzhou, China). ZBE3 cells were transfected with zbTRIM25 siRNA or control siRNA according to the manufacturer's protocol using Lipofectamine 3000 as previously described (18). Twenty-four hours after transfection, ZBE3 cells were infected with RGNNV (MOI = 1) for 24 h and total RNAs were extracted for qRT-PCR analysis.

### Plasmid Construction

The ORF of zbTRIM25 (GenBank accession no. NM200175.1) was sub-cloned into *pCMV-Flag* or *pCMV-Myc* vectors (Invitrogen) to generate recombinant plasmid *pCMV-Flag-zbTRIM25* or *pCMV-Myc-zbTRIM25*, respectively. Full-length zbRIG-I and zbRIG-I deletion mutant cDNAs encoding amino acids 1–187 (zbRIG-I-2CARD), 188–937 (zbRIG-I-Δ2CARD), 812–927 (zbRIG-I-RD), and 188–811 [zbRIG-I-Δ(2CARD+RD)] were inserted into the *pEGFP-N3* vectors. Full-length zbRIG-I was inserted into the *pET-32a(*+*)* (Clontech) vector to generate recombinant plasmid *pET-32a(*+*)-zbRIG-I*. zbTRIM25 deletion mutant zbTRIM25-SPRY and zbTRIM25-ΔSPRY were generated using the *pCMV-Flag-zbTRIM25* plasmid as a template. Primers used for amplifying these genes are listed in Table S1.

HA-K63Ub plasmid was purchased from Rebio (Shanghai, China).

### RNA Isolation and qRT-PCR

RNA extraction and cDNA synthesis were performed using Trizol (Invitrogen) and PrimeScript™ 1st Strand cDNA Synthesis Kit (Takara) according to the manufacturer's instructions. QRT-PCR analyses of *zbTRIM25, zbRIG-I, RNA dependent RNA polymerase* (*RDRP*), RLR signaling pathway related genes (*MAVS, TRAF3, IRF3*, and *IFN 1*), and *ISG15* were performed as previously described (19). Relative expression levels of target genes were normalized to *18s rRNA* using 2^−ΔΔCt^ methods. Data represent the mean ± SD from three independent experiments, each performed in triplicate. Primers sequences used for qRT-PCR are listed in Table S1.

### Dual Luciferase Reporter Assay

HEK 293T cells, pre-seeded in 24-well plates, were transfected with 250 ng of *pGL3-DrIFN 1-pro-Luc* plasmid or *pGL3-Basic* empty vector with 25 ng of *pRL-TK* vector (Promega) together with *pCMV-Myc-zbRIG-I* or *pCMV-Myc* and *pCMV-Flag* or *pCMV-Flag-zbTRIM25* (250 ng per well) for 24 h. Then, cells were incubated with poly I:C for 48 h and lysed. Luciferase activities were measured using the dual-luciferase reporter assay system (Promega). Relative luciferase activities were expressed as the ratio of firefly to Renilla luciferase activity. The results were the representative of three independent experiments in triplicate.

HEK 293T cells, pre-seeded in 24-well plates, were transfected with 250 ng of *pGL3-DrIFN1-pro-Luc* plasmid or *pGL3-Basic* empty vector with 25 ng of *pRL-TK* vector (Promega). Meanwhile, *pCMV-Myc-zbTRIM25*, mutant *zbRIG-I* or empty control plasmids were co-transfected. After being incubated with poly I:C for 48 h, cells were lysed for luciferase assay as described above. At least three independent experiments were performed.

### Immunofluorescence Labeling and Confocal Microscopy

HEK 293T cells, seeded on glass cover slips, were transfected with *pCMV-Myc-zbTRIM25* and *pCMV-Flag-zbRIG-I* plasmids. Twenty-four hours post-transfection, cells were washed with PBS three times and fixed with prechilled methanol and then permeabilized using 1% Triton X-100 in PBS for 10 min and blocked with 5% normal goat serum for 30 min at room temperature (RT). Cells were incubated with anti-Myc and anti-Flag antibodies for 60 min at RT. Finally, cells were washed with PBS and incubated with the appropriate Alexa Fluor 488 or 555 conjugated secondary antibodies for 1 h. After cell nucleus was stained with Hoechst 33342, cells were observed by a confocal microscope (Zeiss, Germany).

### Coimmunoprecipitations (Co-IP) and Western Blotting Analysis

Co-IP and Western blotting experiments were performed as described previously (18). HEK 293T cells in 75-cm^2^ flasks were co-transfected with 10 μg of different plasmid combinations for 48 h. Then, the cells were lysed on ice with lysis buffer for 15 min and were immunoprecipitated with the indicated antibodies. The precipitated samples and whole-cell lysates (Input) were analyzed by immunoblotting with the indicated antibodies.

### His Fusion Protein Expression and Pull-Down Assays

*Escherichia coli* BL21(DE3) cells were transformed with *pET-32a(*+*)-zbRIG-I* or *pET-32a(*+*)* plasmids, respectively. Then, cells were grown in 50 ml of LB medium (Beyotime) containing 0.5 mM isopropyl-1-thio-β-D-galactopyranoside (IPTG) (Sigma) at 18°C overnight with shaking at 120 rpm. Cells were pelleted by centrifugation at 4,500 rpm for 30 min and lysed in 10 ml of lysis buffer (100 mM sodium phosphate, pH 8.0, 600 mM NaCl, and 0.02% Tween-20) (Beyotime) via sonication on ice. The sonicated mixture was centrifuged at 15,000 rpm at 4°C for 20 min, and then the supernatant was affinity-purified with Dynabead His-Tag magnetic beads (Invitrogen) according to the manufacturer's instruction.

His pull-down assays were performed as described previously with some modifications (20). His-zbRIG-I-magnetic beads were incubated with the lysates of HEK 293T cells transfected with *pCMV-Flag-zbTRIM25* or *pCMV-Flag* empty vectors on a roller, respectively. After incubation at 4°C overnight, the magnetic beads were washed three times with lysis buffer to remove unbound His-zbRIG-I and then analyzed via Western blotting using anti-Flag or anti-His antibodies. His tag protein alone was served as a negative control.

### Ubiquitination Assays

Ubiquitination assays were performed as described previously with some modifications (21). HEK 293T cells, pre-seeded in 75-cm^2^ flasks overnight, were co-transfected with 10 μg of different plasmid combinations. Cells were lysed at 24 h after transfection, and then GFP-zbRIG-I mutants were immunoprecipitated with anti-GFP antibodies as described above. Immunoprecipitates or Input were analyzed by immunoblotting with the indicated antibodies.

### Statistics Analysis

All statistics were calculated using SPSS version 20. Differences between control and treatment groups were assessed by one-way ANOVA. *P* < 0.05 is considered statistically significantly different. *P* < 0.01 was considered highly significant.

## Results

### zbTRIM25 Expression Is Down-Regulated During RGNNV Infection *in vitro* and *in vivo*

**Figure 1:**
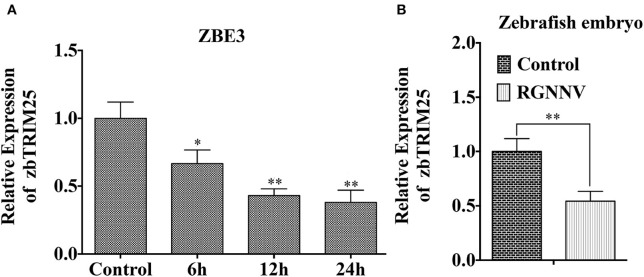
zbTRIM25 expression is inhibited in zebrafish during RGNNV infection. **(A)** ZBE3 cells were infected with RGNNV for the indicated time points. **(B)** Zebrafish embryos were infected with RGNNV for 24 h. The expression of *zbTRIM25* mRNA was tested by qRT-PCR and normalized with *18s rRNA*. Asterisks indicate significant differences between groups (*p < 0.05; **p < 0.01).

As shown in Figure 1A, mRNA level of *zbTRIM25* was down-regulated within 24 h after RGNNV infection. Meanwhile, we also investigated the expression of *zbTRIM25* in RGNNV-infected zebrafish embryos at 24 h, and the results were concordant with ZBE3 cells (Figure 1B). These data indicated a potential role of zbTRIM25 in innate immune response to RGNNV infection.

### zbTRIM25 Positively Regulates RLR Signaling Pathway in Zebrafish

**Figure 2:**
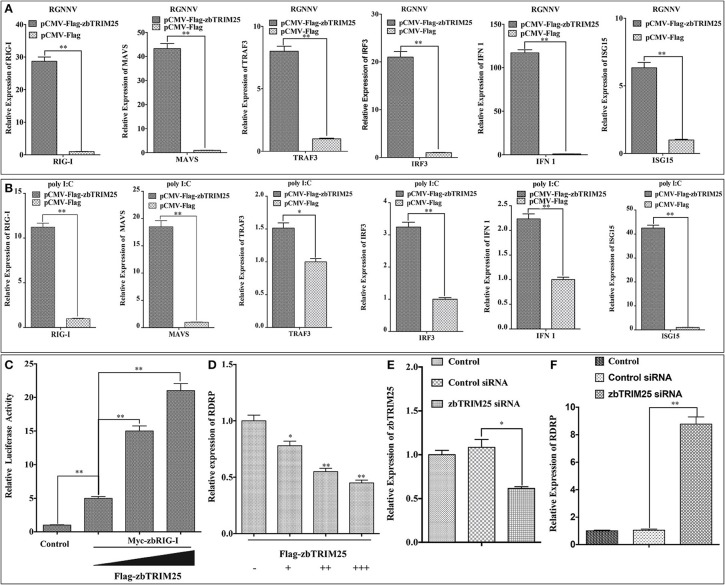
zbTRIM25 potentiates RLR signaling pathway. **(A,B)** ZBE3 cells were transfected with *pCMV-Flag-zbTRIM25* or *pCMV-Flag* vector for 24 h. After treated with RGNNV **(A)** or poly I:C **(B)**, the transcript levels of *RIG-I, MAVS, TRAF3, IRF3, IFN 1*, and *ISG15* mRNA in ZBE3 cells were analyzed. **(C)** HEK 293T cells were transfected with *pCMV-Myc* or *pCMV-Myc-zbRIG-I* together with *pCMV-Flag* or increasing amount of *pCMV-Flag-zbTRIM25* as well as *DrIFN1 pro-Luc* and *pRL-TK*. Luciferase activities were measured and normalized to the amount of Renilla Luciferase activities. **(D)** ZBE3 cells were transfected with *pCMV-Flag* and increasing amount of *pCMV-Flag-zbTRIM25* for 24 h, and then infected with RGNNV for 24 h. QRT-PCR analysis was performed for *RDRP*. **(E,F)** ZBE3 cells were either not transfected (Control) or transfected with 100 nM Control siRNA (NC) or 100 nM zbTRIM25 siRNA. Cells were then subcultured for 24 h and infected with RGNNV for 24 h. Cells were harvested subsequently. Levels of *zbTRIM25* and *RDRP* mRNA were analyzed by qRT-PCR and normalized with *18s rRNA*. Data represent the mean + SD (*n* = 3). Asterisks indicate significant differences between groups (*p < 0.05; **p < 0.01).

In mammals, TRIM25 has been suggested to promote IFN-β production by functioning as a key upstream activator of RIG-I to activate the RLR signaling pathway (10). To investigate whether zbTRIM25 regulated RLR signaling pathway in zebrafish, the expression of several RLR signaling pathway-related genes was measured in zbTRIM25 overexpressing ZBE3 cells. As shown in Figure 2A, overexpression of zbTRIM25 markedly enhanced the expression of *RIG-I, MAVS, TRAF3, IRF3, IFN 1*, and *ISG15* during RGNNV infection. Similar results were detected in zbTRIM25 overexpressing ZBE3 cells treated with poly I:C (Figure 2B). Furthermore, co-expression of zbTRIM25 with zbRIG-I induced a dose-dependent increase in IFN activation compared with the zbRIG-I overexpression alone (Figure 2C). Overexpression of zbTRIM25 dose-dependently inhibited RGNNV replication (Figure 2D). On the contrary, knockdown of zbTRIM25 using siRNA increased the level of *RDRP* in RGNNV-infected ZBE3 cells (Figures 2E,F). All these results demonstrate that zbTRIM25 is a positive regulator of RLR signaling pathway and functions as an antiviral factor during RGNNV infection in zebrafish.

### zbTRIM25 Interacts With zbRIG-I

**Figure 3:**
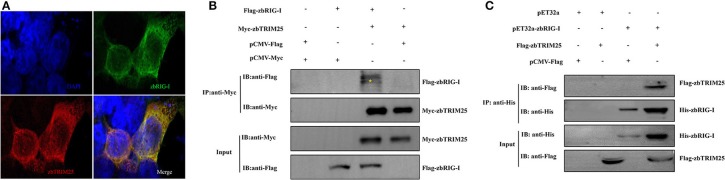
zbTRIM25 interacts with zbRIG-I. **(A)** HEK 293T cells were transfected with *pCMV-Flag-zbRIG-I* and *pCMV-Myc-zbTRIM25* plasmids for 24 h, and then cells were immunostained with anti-Myc and anti-Flag antibodies and analyzed by fluorescence microscopy. All nuclei were stained with Hoechst 33342. **(B)** HEK 293T cells were transfected with plasmids as indicated for 24 h. After treated with poly I:C for 24 h, cells were lysed, and the cell lysates were either analyzed directly by using anti-Myc and anti-Flag antibodies via Western blotting (Input) or subjected to immunoprecipitation using anti-Myc antibodies. The precipitates (IP) were analyzed by Western blotting with anti-Myc and anti-Flag antibodies, respectively. **(C)** The purified His-zbRIG-I proteins were incubated with the lysates of HEK 293T cells transfected with the indicated plasmids, respectively. The pulled down proteins and original cell lysates were examined by Western blotting with indicated antibodies.

To elucidate the mechanism by which zbTRIM25 participates in RLR signaling pathway in zebrafish, the interaction of zbTRIM25 and zbRIG-I was investigated. We co-expressed *Myc-zbTRIM25* and *Flag-zbRIG-I* plasmids in HEK 293T cells, and immunofluorescence imaging showed zbTRIM25 and zbRIG-I colocalized in the cytoplasm of HEK293T cells (Figure 3A). Co-IP against the Myc tag revealed that zbTRIM25 could interact with full-length zbRIG-I but not with Flag-vector (Figure 3B). His pull-down analysis showed that zbTRIM25 was directly bound to zbRIG-I (Figure 3C). All these data suggest that zbTRIM25 interacts with zbRIG-I.

### SPRY Domain of zbTRIM25 Interacts With 2CARD and RD Regions of zbRIG-I

**Figure 4:**
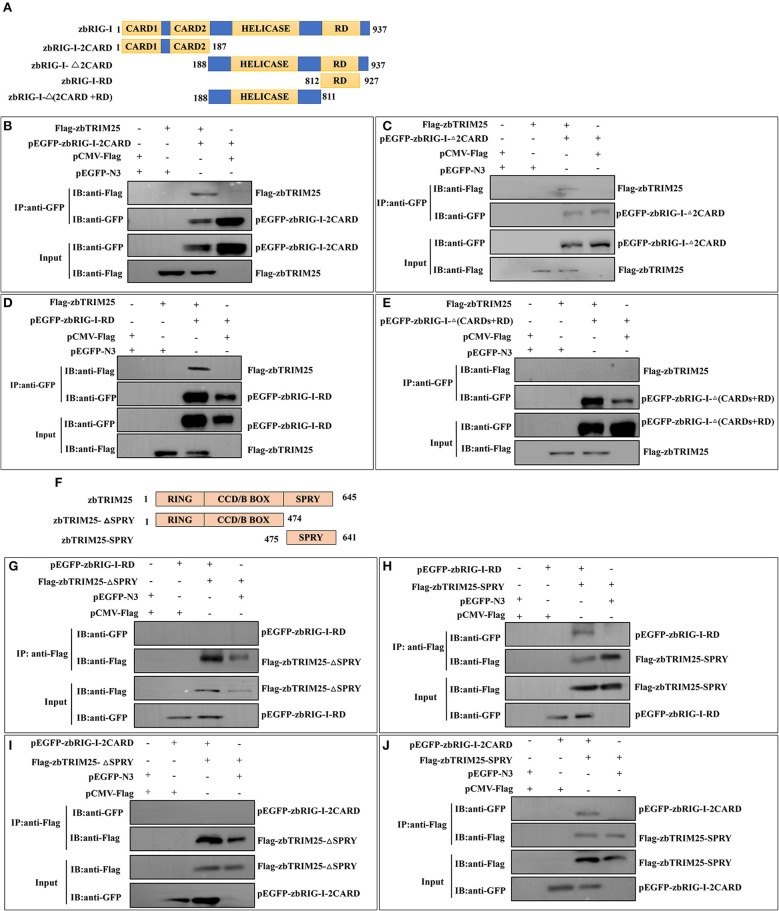
Physical interaction of zbTRIM25 with zbRIG-I. **(A)** Schematic representation of full-length zbRIG-I and zbRIG-I deletion mutants. **(B–E)** Interactions of GFP-tagged zbRIG-I-2CARD **(B)**, zbRIG-I-Δ2CARD **(C)**, zbRIG-I-RD **(D)**, or zbRIG-I-Δ(CARDs+RD) **(E)** with Flag-tagged zbTRIM25 were examined using immunoprecipitation assays. HEK 293T cells were transfected with plasmids as indicated for 24 h. After treated with poly I:C for 24 h, cells were lysed, and the cell lysates were either analyzed directly by using anti-GFP and anti-Flag antibodies via Western blotting (Input) or subjected to immunoprecipitation using anti-GFP antibodies. The precipitates (IP) were analyzed by Western blotting with anti-GFP and anti-Flag antibodies, respectively. **(F)** Schematic illustration of zbTRIM25 truncations. Interactions of GFP-zbRIG-I-RD with Flag-zbTRIM25-ΔSPRY **(G)** or Flag-zbTRIM25-SPRY **(H)**, GFP-zbRIG-I-2CARD with Flag-zbTRIM25-ΔSPRY **(I)**, or Flag-zbTRIM25-SPRY **(J)** were examined using immunoprecipitation assays. HEK 293T cells were transfected with plasmids as indicated for 24 h. Cells were lysed after treated with poly I:C for 24 h. Immunoprecipitation and immunoblotting were performed with indicated antibodies.

To identify the region involved in the zbRIG-I/zbTRIM25 interaction, firstly, zbRIG-I deletion mutants [*pEGFP-zbRIG-I-2CARD, pEGFP-zbRIG-I-*Δ*2CARD, pEGFP-RIG-I-RD*, and *pEGFP-RIG-I-*Δ(*2CARD*+*RD*)] were constructed and co-transfected with *Flag-zbTRIM25* in HEK 293T cells (Figure 4A). zbRIG-I-2CARD, zbRIG-I-Δ2CARD, and zbRIG-I-RD could bind to zbTRIM25 individually (Figures 4B–D); however, zbRIG-I-Δ(2CARD+RD) failed to co-precipitate with zbTRIM25 (Figure 4E). These results indicate that zbRIG-I binds to zbTRIM25 through its N-terminal 2CARD region and the C-terminal RD region. Furthermore, we constructed two truncations of zbTRIM25 (zbTRIM25-SPRY and zbTRIM25-ΔSPRY) co-transfected with zbRIG-I-2CARD or zbRIG-I-RD in HEK 293T cells, respectively (Figure 4F). We found that the SPRY domain of zbTRIM25 interacted with 2CARD and RD regions of zbRIG-I (Figures 4G–J). Collectively, these results indicate that the SPRY domain of zbTRIM25 is responsible for its interaction with 2CARD and RD regions of zbRIG-I.

### zbTRIM25 Ubiquitinates Both 2CARD and RD Regions of zbRIG-I

**Figure 5:**
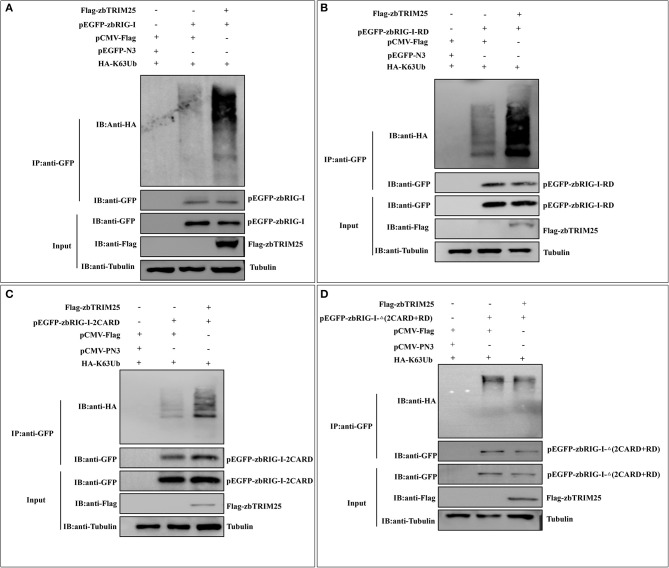
zbTRIM25 promotes zbRIG-I ubiquitination. **(A–D)** HEK 293T cells were transfected with plasmids as indicated for 24 h. At 24 h after poly I:C treatment, cells were lysed, and the cell lysates were either analyzed directly by using anti-GFP, anti-Flag, and anti-tubulin antibodies via Western blotting (Input) or subjected to immunoprecipitation using anti-GFP antibodies. The precipitates (IP) were analyzed by Western blotting with anti-GFP and anti-HA antibodies, respectively.

To investigate whether the E3 ligase activity of zbTRIM25 is involved in the regulation of zbRIG-I, the ubiquitination of zbRIG-I was tested in zbTRIM25 overexpressing cells. We found that zbTRIM25 markedly promoted the K63 polyubiquitination of zbRIG-I (Figure 5A). Furthermore, HEK 293T cells were transfected with Flag-tagged zbTRIM25, zbRIG-I deletion mutants [*GFP-zbRIG-I-2CARD, GFP-zbRIG-I-*Δ(*2CARD*+*RD*), and *GFP-zbRIG-I-RD*], and HA-tagged K63 ubiquitin, and our results showed that zbTRIM25 obviously enhanced the ubiquitination of zbRIG-I-2CARD and zbRIG-I-RD (Figures 5B,C), but not zbRIG-I-Δ(2CARD+RD) (Figure 5D). These data suggest that zbTRIM25 ubiquitinates both N-terminal 2CARD and C-terminal RD regions of zbRIG-I.

### zbTRIM25-Mediated Ubiquitination of zbRIG-I 2CARD and RD Regions Is Important for IFN Inducing

**Figure 6:**
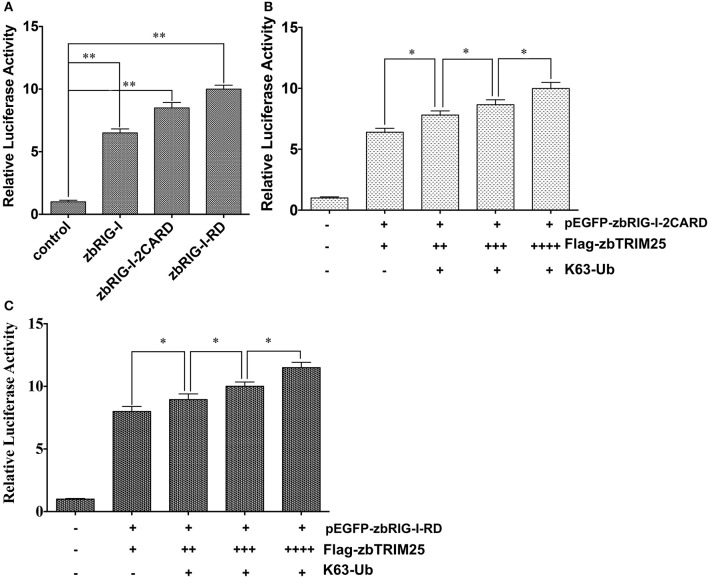
zbTRIM25-mediated K63 ubiquitination in 2CARD and RD regions of zbRIG-I is important for IFN induction. **(A)** HEK 293T cells were transfected with *pEGFP-zbRIG-I, pEGFP-zbRIG-I-2CARD*, and *pEGFP-zbRIG-I-RD* as well as *DrIFN1 pro-Luc* and *pRL-TK* vectors. **(B,C)** HEK 293T cells were transfected with *pEGFP-zbRIG-I-2CARD* or *pEGFP-zbRIG-I-RD* together with *HA-K63Ub* and increasing amount of *pCMV-Flag-zbTRIM25* as well as *DrIFN1 pro-Luc* and *pRL-TK* plasmids. Luciferase activities were measured and normalized to the amount of Renilla Luciferase activities. Data represent the mean + SD (*n* = 3). Asterisks indicate significant differences between groups (*p < 0.05; **p < 0.01).

It has been reported that ubiquitination of RIG-I by TRIM25 is vital for IFN signaling. Thus, the effect of zbTRIM25-mediated zbRIG-I ubiquitination on zbRIG-I's IFN-inducing activities was assessed. Our results showed that ectopic expression of zbRIG-I-2CARD and zbRIG-I-RD could enhance IFN promoter activity (Figure 6A), and this activation was markedly enhanced by zbTRIM25 overexpression (Figures 6B,C). Furthermore, overexpression of K63-linked ubiquitin dose-dependently increased the promotion effect of zbTRIM25 on zbRIG-I-2CARD and RD mediated IFN 1 promoter activation (Figures 6B,C). These data confirm the importance of zbTRIM25-mediated K63 ubiquitination in the N-terminal 2CARD region and C-terminal RD region of zbRIG-I for zbRIG-I-mediated IFN induction.

## Discussion

RLR signaling pathway plays crucial roles in recognizing viral infections and initiating the antiviral immune response. RIG-I, as an important component of RLR signaling pathway, can detect viral dsRNAs in the cytoplasm and induce type I IFN production and the secretion of pro-inflammatory cytokines to suppress virus spread during virus infection (22). Multiple studies have demonstrated that the ubiquitination of RIG-I plays an important role in the RIG-I-mediated antiviral signaling pathway. For instance, TRIM25, TRIM4, and MEX3C positively regulate RIG-I-mediated signaling by targeting RIG-I for K63-linked polyubiquitination (23, 24). TRIM25, well-known as an ubiquitin E3 ligase and an ISG15 E3 ligase, is widely involved in the regulation of innate immunity (10, 25). In mammals, previous reports showed that TRIM25 enhanced RLRs antiviral pathway by binding viral RNA-activated RIG-I to induce its K63-linked polyubiquitination and subsequent IFNs and ISGs production (26). In teleost fish, several TRIM25 homologs were reported to play a pivotal role in innate immunity (14, 15); however, the mechanisms by which fish TRIM25 modulates the innate immune response against viruses remain elusive. Here, we found that zbTRIM25 positively regulated RLR signaling pathway and facilitated zbRIG-I-mediated IFN 1 promoter activation, and overexpression of zbTRIM25 inhibited RGNNV infection, indicating the conservative antiviral properties of TRIM25 in fish and mammals.

Several reports showed that TRIM25 was involved in the regulation of antiviral innate immunity by targeting RIG-I (10, 27). The mammal RIG-I protein contains two N-terminal CARD-like domains, a C-terminal RD region and an RNA helicase region (28). In zebrafish, RIG-Ia (an insertion variant of RIG-I) and RIG-Ib (the typical RIG-I) were identified as two transcripts of RIG-I, and overexpression of RIG-Ib in cultured fish cells, but not RIG-Ia, activated zebrafish type I IFN and induced antiviral response (29). Thus, in this report, we investigated the interaction of zbTRIM25 and zbRIG-I (RIG-Ib), and our results showed that zbTRIM25 was directly associated with zbRIG-I and especially the 2CARD or RD region of zbRIG-I was sufficient for its interaction with zbTRIM25. TRIM25 is characterized by an N-terminal region containing a catalytic RING domain, one or two B-box domains, a coiled-coil dimerization domain, and a C-terminal SPRY domain (30). Among these domains, SPRY was associated with protein–protein interactions and/or RNA binding (31). Gack et al. reported that the C-terminal SPRY domain of TRIM25 interacted with the first CARD of RIG-I, but not the helicase region and RD of RIG-I, and this interaction delivered the K63-linked ubiquitin moieties to the second CARD of RIG-I, which facilitated the dimerization of RIG-I and subsequent interaction with MAVS to induce antiviral signal transduction (10). We further investigate whether the SPRY domain of zbTRIM25 was responsible for its interaction with zbRIG-I. Unlike previous studies, we found that the SPRY domain of zbTRIM25 interacted not only with 2CARD but also with RD regions of zbRIG-I. In non-infected cells, RD covered the RNA-binding and helicase domains and CARDs folded over one another, which made RIG-I to exist in an auto-repressed conformation. Upon virus infection, viral RNAs interacted with the RD and the helicase domain of RIG-I, which in turn exposed the CARDs for MAVS interaction, thereby triggering antiviral responses (32, 33). Considering the interaction between RD of RIG-I and viral RNAs, we speculated that the interaction of zbTRIM25 and zbRIG-I RD might inhibit zbRIG-I sensing of viral RNAs. Meanwhile, it has been known that CARD domains of RIG-I are widely involved in its interaction with other proteins, such as MAVS, TRIM40, and virus proteins (27, 34, 35). Thus, the interaction of zbTRIM25 and zbRIG-I RD might also make room for other proteins to bind to 2CARD of zbRIG-I, zbTRIM25, and other proteins and will work cooperatively in regulation of RLR signaling pathway. The differences between the findings for zbTRIM25 and TRIM25 in mammals indicate that zbTRIM25 may regulate RLR signaling pathway in various ways.

Ubiquitination is a vital post-translational modification for the modulation of RIG-I activity. Several E3 ubiquitin ligases that mediate K63-linked ubiquitination of RIG-I for its activation have been identified. For instance, MEX3C overexpression caused the K63-linked ubiquitination of RIG-I-2CARD but not RIG-I-Δ2CARD, and lysines 48, 99, and 169 of RIG-I were required for RIG-I ubiquitination by MEX3C (23). RNF135 mediated the K63-linked polyubiquitination of RIG-I-RD, and lysines 849 and 851 residues of RIG-I were crucial for RNF135-mediated ubiquitination (36). In contrast to RNF135, TRIM25 mediated the K63-linked polyubiquitination of RIG-I-2CARD, but not RIG-I-Δ2CARD, and the lysine 172 residue of RIG-I was critical for efficient TRIM25-mediated ubiquitination and the ability of RIG-I to activate antiviral signal transduction (10). Our results indicated that zbTRIM25 mediated K63-linked polyubiquitination of both 2CARD and RD regions of zbRIG-I, which is distinct from the findings in mammals that TRIM25 only targeted and promoted the K63-linked polyubiquitination of RIG-I 2CARD. In addition, our reporter analysis showed that overexpression of zbRIG-I-2CARD led to the activation of IFN 1 promoter, which is similar with other reports (37). Overexpression of zbRIG-I-RD also resulted in the activation of IFN 1 promoter. Furthermore, K63-linked ubiquitin is essential for the zbTRIM25-mediated enhancement of zbRIG-I 2CARD and RD-dependent IFN 1 promoter activation. zbRIG-I possessed capacities in the induction of IFNs and ISGs to enhance the antiviral response (38). Taken together, these findings suggest that zbTRIM25-mediated ubiquitination of 2CARD and RD regions of zbRIG-I is crucial for its antiviral innate immune response. However, due to the lack of TRIM25 or RIG-I-knockout zebrafish, we cannot assess the impact of the zebrafish TRIM25/RIG-I pathway at the *in vivo* level. A recent study demonstrated that zebrafish RNF135 also interacted with and ubiquitinated zbRIG-I (39). Further studies will be performed to determine the precise architecture of the zebrafish TRIM25/RNF135/RIG-I protein complex and the mechanism by which zbTRIM25 and zbRNF135 worked together to regulate ubiquitination of zbRIG-I.

It was known that several virus proteins could positively or negatively regulate RLR signaling pathway by targeting its key components or regulatory proteins (22). For instance, paramyxovirus V proteins interacted with the RIG-I/TRIM25 regulatory complex and inhibited RIG-I signaling (27). Influenza A virus NS1 protein bound to TRIM25 to block ubiquitination of the RIG-I (40). Severe acute respiratory syndrome nucleocapsid inhibited TRIM25-mediated RIG-I ubiquitination, causing the inhibition of IFN production (41). The RGNNV genome encodes a structural (capsid protein, CP) and a nonstructural (RNA-dependent RNA polymerase, RdRp) protein (3). Huang et al. reported that RDRP from OGNNV induced IFN by activating IRF3, the key regulatory component of RLRs-IFN signaling (42), indicating that RDRP might be a positive RLR signaling pathway. Whether RDRP targets the key components of RLR signaling pathway to exert its positive regulation role is a question that deserves further research. In addition, some miRNAs could target critical regulatory proteins of RLR pathway for immune evasion (43, 44); whether RGNNV infection-related miRNA was also involved in the regulation of RLR signaling pathway needs to be further investigated.

**Figure 7:**
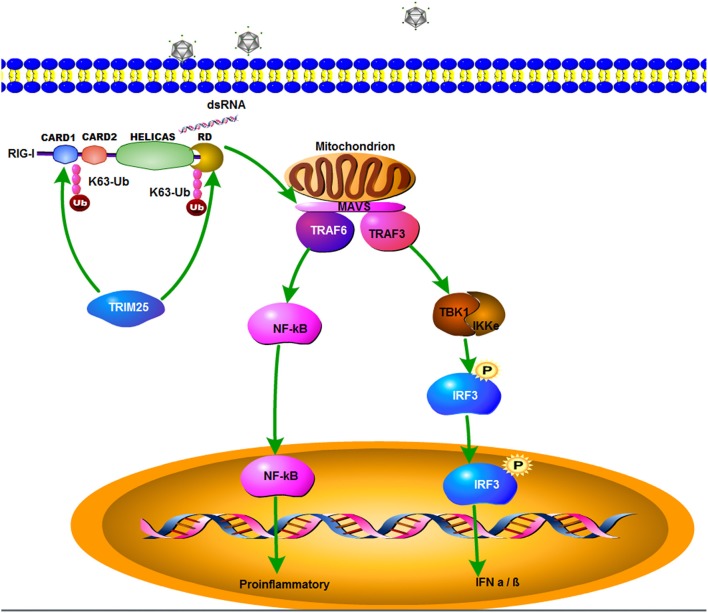
A schematic model of zbTRIM25-mediated RLR signaling pathway during RGNNV infection. zbTRIM25 interacts with and catalyzes the K63 polyubiquitination of 2CARD and RD regions of zbRIG-I, which subsequently induces the activation of downstream signaling event via MAVS, and thereby inhibits viral infection.

In summary, zbTRIM25 is identified as a positive regulator of RLR signaling pathway by targeting zbRIG-I. The SPRY domain of zbTRIM25 is required for its interaction with 2CARD and RD regions of zbRIG-I. zbTRIM25 promotes K63 polyubiquitination of both zbRIG-I 2CARD and RD regions, which subsequently induces the activation of downstream signaling event via MAVS and thereby inhibits viral infection (Figure 7). These findings represent a new mechanism underlying the regulation of RLR signaling pathway.

## Data Availability Statement

All datasets generated for this study are included in the article/Supplementary Material.

## Ethics Statement

The animal study was reviewed and approved by the Ethics Committee of Sun Yat-Sen University.

## Author Contributions

YJ and KJ performed all experiments with assistance from YX, WZ, PJ, and WL analyzed data. KJ and MY conceived the study and designed experiments. KJ and MY wrote the manuscript. All authors read and approved the final manuscript.

### Conflict of Interest

The authors declare that the research was conducted in the absence of any commercial or financial relationships that could be construed as a potential conflict of interest.

## References

[1] Dolasia K Bisht MK Pradhan G Udgata A Mukhopadhyay S. TLRs/NLRs: Shaping the landscape of host immunity. Int Rev Immunol. (2018) 37:3–19. 10.1080/08830185.2017.1397656 29193992

[2] Eisenacher K Krug A. Regulation of RLR-mediated innate immune signaling - It is all about keeping the balance. Eur J Cell Biol. (2012) 91:36–47. 10.1016/j.ejcb.2011.01.011 21481967

[3] Costa JZ Thompson KD. Understanding the interaction between Betanodavirus and its host for the development of prophylactic measures for viral encephalopathy and retinopathy. Fish Shellfish Immun. (2016) 53:35–49. 10.1016/j.fsi.2016.03.033 26997200

[4] Chen HY Liu WT Wu SY Chiou PP Li YH Chen YC. RIG-I specifically mediates group II type I IFN activation in nervous necrosis virus infected zebrafish cells. Fish Shellfish Immun. (2015) 43:427–35. 10.1016/j.fsi.2015.01.012 25634257

[5] Jia P Jia KT Chen LM Le Y Jin YL Zhang J. Identification and characterization of the melanoma differentiation - associated gene 5 in sea perch, *Lateolabrax japonicus*. Dev Comp Immunol. (2016) 61:161–8. 10.1016/j.dci.2016.03.029 27039216

[6] Jia P Zhang J Jin YL Zeng L Jia KT Yi MS. Characterization and expression analysis of laboratory of genetics and physiology 2 gene in sea perch, *Lateolabrax japonicus*. Fish Shellfish Immun. (2015) 47:214–20. 10.1016/j.fsi.2015.09.004 26363231

[7] Wang WD Jiang MH Liu S Zhang SK Liu W Ma YW. RNF122 suppresses antiviral type I interferon production by targeting RIG-I CARDs to mediate RIG-I degradation. Proc Natl Acad Sci USA. (2016) 113:9581–6. 10.1073/pnas.1604277113 27506794 PMC5003265

[8] Narayan K Waggoner L Pham ST Hendricks GL Waggoner SN Conlon J. TRIM13 is a negative regulator of MDA5-mediated type I interferon production. J Virol. (2014) 88:10748–57. 10.1128/JVI.02593-13 25008915 PMC4178852

[9] Zhong B Zhang Y Tan B Liu TT Wang YY Shu HB. The E3 ubiquitin ligase RNF5 targets virus-induced signaling adaptor for ubiquitination and degradation. J Immunol. (2010) 184:6249–55. 10.4049/jimmunol.0903748 20483786

[10] Gack MU Shin YC Joo CH Urano T Liang C Sun LJ. TRIM25 RING-finger E3 ubiquitin ligase is essential for RIG-I-mediated antiviral activity. Nature. (2007) 446:916–2. 10.1038/nature05732 17392790

[11] Martin-Vicente M Medrano LM Resino S Garcia-Sastre A Martinez I. TRiM25 in the regulation of the antiviral innate immunity. Front Immunol. (2017) 8:1187. 10.3389/fimmu.2017.01187 29018447 PMC5614919

[12] Nguyen NTH Now H Kim WJ Kim N Yoo JY. Ubiquitin-like modifier FAT10 attenuates RIG-I mediated antiviral signaling by segregating activated RIG-I from its signaling platform. Sci Rep. (2016) 6:23377. 10.1038/srep23377 26996158 PMC4800306

[13] Kong HJ Lee YJ Shin J Cho HK Kim WJ Kim HS. Molecular characterization of tripartite motif protein 25 (TRIM25) involved in ERα-mediated transcription in the Korean rose bitterling *Rhodeus uyekii*. Comp Biochem Physiol B Biochem Mol Biol. (2012) 163:147–53. 10.1016/j.cbpb.2012.05.015 22642868

[14] Yang Y Huang YH Yu YP Yang M Zhou S Qin QW. RING domain is essential for the antiviral activity of TRIM25 from orange spotted grouper. Fish Shellfish Immun. (2016) 55:304–14. 10.1016/j.fsi.2016.06.005 27276113

[15] Zhou ZZ Wei K Zhang JS. The two TRIM25 isoforms were differentially induced in *Larimichthys crocea* post poly (I:C) stimulation. Fish Shellfish Immun. (2019) 86:672–9. 10.1016/j.fsi.2018.12.009 30529437

[16] Kimmel CB Ballard WW Kimmel SR Ullmann B Schilling TF. Stages of embryonic development of the zebrafish. Dev Dyn. (1995) 203:253–310. 10.1002/aja.1002030302 8589427

[17] Jin YL Chen LM Le Y Li YL Hong YH Jia KT. Establishment of a cell line with high transfection efficiency from zebrafish *Danio rerio* embryos and its susceptibility to fish viruses. J Fish Biol. (2017) 91:1018–31. 10.1111/jfb.13387 28833122

[18] Jia KT Wu YY Liu ZY Mi S Zheng YW He J. Mandarin fish caveolin 1 interaction with major capsid protein of infectious spleen and kidney necrosis virus and its role in early stages of infection. J Virol. (2013) 87:3027–38. 10.1128/JVI.00552-12 23283951 PMC3592132

[19] Zhang WW Li ZL Jia P Liu W Yi MS Jia KT. Interferon regulatory factor 3 from sea perch (*Lateolabrax japonicus*) exerts antiviral function against nervous necrosis virus infection. Dev Comp Immunol. (2018) 88:200–5. 10.1016/j.dci.2018.07.014 30016710

[20] Liu XF Wang XY Wang Q Luo MY Guo HC Gong WJ. The eukaryotic translation initiation factor 3 subunit E binds to classical swine fever virus NS5A and facilitates viral replication. Virology. (2018) 515:11–20. 10.1016/j.virol.2017.11.019 29223786

[21] Wu HS Shi LY Zhang YN Peng XR Zheng TY Li YH. Ubiquitination is essential for avibirnavirus replication by supporting VP1 polymerase activity. J Virol. (2019) 93:e01899–18. 10.1128/JVI.01899-18 30429342 PMC6340032

[22] Chan YK Gack MU. RIG-I-like receptor regulation in virus infection and immunity. Curr Opin Virol. (2015) 12:7–14. 10.1016/j.coviro.2015.01.004 25644461 PMC5076476

[23] Kuniyoshi K Takeuchi O Pandey S Satoh T Iwasaki H Akira S. Pivotal role of RNA-binding E3 ubiquitin ligase MEX3C in RIG-I-mediated antiviral innate immunity. Proc Natl Acad Sci USA. (2014) 111:5646–51. 10.1073/pnas.1401674111 24706898 PMC3992669

[24] Yan J Li Q Mao AP Hu MM Shu HB. TRIM4 modulates type I interferon induction and cellular antiviral response by targeting RIG-I for K63-linked ubiquitination. J Mol Cell Biol. (2014) 6:154–63. 10.1093/jmcb/mju005 24755855

[25] Pauli EK Chan YK Davis ME Gableske S Wang MK Feister KF. The ubiquitin-specific protease USP15 promotes RIG-I-mediated antiviral signaling by deubiquitylating TRIM25. Sci Signal. (2014) 7:ra3. 10.1126/scisignal.2004577 24399297 PMC4008495

[26] Sanchez JG Chiang JJ Sparrer KMJ Alam SL Chi M Roganowicz MD. Mechanism of TRIM25 catalytic activation in the antiviral RIG-I pathway. Cell Rep. (2016) 16:1315–25. 10.1016/j.celrep.2016.06.070 27425606 PMC5076470

[27] Sanchez-Aparicio MT Feinman LJ Garcia-Sastre A Shaw ML. Paramyxovirus V proteins interact with the RIG-I/TRIM25 regulatory complex and inhibit RIG-I signaling. J Virol. (2018) 92:e01960–17. 10.1128/JVI.01960-17 29321315 PMC5827389

[28] Lee MK Kim HE Park EB Lee J Kim KH Lim K. Structural features of influenza A virus panhandle RNA enabling the activation of RIG-I independently of 5'-triphosphate. Nucleic Acids Res. (2016) 44:8407–16. 10.1093/nar/gkw525 27288441 PMC5041458

[29] Zou PF Chang MX Li Y Zhang SH Fu JP Chen SN. Higher antiviral response of RIG-I through enhancing RIG-I/MAVS-mediated signaling by its long insertion variant in zebrafish. Fish Shellfish Immun. (2015) 43:13–24. 10.1016/j.fsi.2014.12.001 25524497

[30] Meroni G Diez-Roux G. TRIM/RBCC, a novel class of 'single protein RING finger' E3 ubiquitin ligases. Bioessays. (2005) 27:1147–57. 10.1002/bies.20304 16237670

[31] D'Cruz AA Babon JJ Norton RS Nicola NA Nicholson SE. Structure and function of the SPRY/B30.2 domain proteins involved in innate immunity. Protein Sci. (2013) 22:1–10. 10.1002/pro.2185 23139046 PMC3575854

[32] Leung DW Amarasinghe GK. Structural insights into RNA recognition and activation of RIG-I-like receptors. Curr Opin Struc Biol. (2012) 22:297–303. 10.1016/j.sbi.2012.03.011 22560447 PMC3383332

[33] Lu C Xu HY Ranjith-Kumar CT Brooks MT Hou TY Hu F. The structural basis of 5 ' triphosphate double-stranded RNA recognition by RIG-I C-terminal domain. Structure. (2010) 18:1032–43. 10.1016/j.str.2010.05.007 20637642 PMC2919622

[34] Zhao C Jia M Song H Yu Z Wang W Li Q. The E3 ubiquitin ligase TRIM40 attenuates antiviral immune responses by targeting MDA5 and RIG-I. Cell Rep. (2017) 21:1613–23. 10.1016/j.celrep.2017.10.020 29117565

[35] Seth RB Sun L Ea CK Chen ZJ. Identification and characterization of MAVS, a mitochondrial antiviral signaling protein that activates NF-kappaB and IRF 3. Cell. (2005) 122:669–82. 10.1016/j.cell.2005.08.012 16125763

[36] Oshiumi H Matsumoto M Hatakeyama S Seya T. Riplet/RNF135, a RING finger protein, ubiquitinates RIG-I to promote interferon-beta induction during the early phase of viral infection. J Biol Chem. (2009) 284:807–17. 10.1074/jbc.M804259200 19017631

[37] Nie L Zhang YS Dong WR Xiang LX Shao JZ. Involvement of zebrafish RIG-I in NF-kappaB and IFN signaling pathways: insights into functional conservation of RIG-I in antiviral innate immunity. Dev Comp Immunol. (2015) 48:95–101. 10.1016/j.dci.2014.09.008 25265425

[38] Chen SN Zou PF Nie P. Retinoic acid-inducible gene I (RIG-I)-like receptors (RLRs) in fish: current knowledge and future perspectives. Immunology. (2017) 151:16–25. 10.1111/imm.12714 28109007 PMC5382327

[39] Lai YX Liang M Hu L Zeng ZC Lin H Yi G. RNF135 is a positive regulator of IFN expression and involved in RIG-I signaling pathway by targeting RIG-I. Fish Shellfish Immun. (2019) 86:474–9. 10.1016/j.fsi.2018.11.070 30508673

[40] Gack MU Albrecht RA Urano T Inn KS Huang IC Carnero E. Influenza A virus NS1 targets the ubiquitin ligase TRIM25 to evade recognition by the host viral RNA sensor RIG-I. Cell Host Microbe. (2009) 5:439–49. 10.1016/j.chom.2009.04.006 19454348 PMC2737813

[41] Hu Y Li W Gao T Cui Y Jin Y Li P. The severe acute respiratory syndrome coronavirus nucleocapsid inhibits Type I interferon production by interfering with TRIM25-mediated RIG-I ubiquitination. J Virol. (2017) 91:e02143–6. 10.1128/JVI.02143-16 28148787 PMC5375661

[42] Huang R Zhou Q Shi Y Zhang J He J Xie J. Protein A from orange-spotted grouper nervous necrosis virus triggers type I interferon production in fish cell. Fish Shellfish Immunol. (2018) 79:234–43. 10.1016/j.fsi.2018.05.006 29733958

[43] Xu C He X Zheng Z Zhang Z Wei C Guan K. Downregulation of microRNA miR-526a by enterovirus inhibits RIG-I-dependent innate immune response. J Virol. (2014) 88:11356–68. 10.1128/JVI.01400-14 25056901 PMC4178780

[44] Hou J Wang P Lin L Liu XG Ma F An HZ. MicroRNA-146a feedback inhibits RIG-I-dependent Type I IFN production in macrophages by targeting TRAF6, IRAK1, and IRAK2. J Immunol. (2009) 183:2150–8. 10.4049/jimmunol.0900707 19596990

