# Supplementary material for: Zebrafish TRIM25 Promotes Innate Immune Response to RGNNV Infection by Targeting 2CARD and RD Regions of RIG-I for K63-Linked Ubiquitination
Source: Front Immunol. 2019 Dec 3;10:2805. doi: 10.3389/fimmu.2019.02805 (PMC6901795; doi:10.3389/fimmu.2019.02805)
Supplement: Supplementary file 1 [file Data_Sheet_1.pdf]

Supplementary Material

1 Supplementary Tables

Table S1. Primers used in this study

Primer name	Sequences (5'-3')	Comment
18s rRNA-F	TCGCTAGTTGGCATCGTTTATG	QPCR of 18s rRNA
18s rRNA-R	CGGAGGTTCGAAGACGATCA	QPCR of 18s rRNA
NNV-RDRP-F	GCTTTATGCGTGAGTGCGTC	QPCR of RDRP
NNV-RDRP-R	GCTGTTTCCGTCTGTTGTGAG	QPCR of RDRP
zbTRIM25-Myc-XhoI-F	CCGCTCGAGGTGCGGAACAAATGTCTCTG	pCMV-Myc-zbTRIM25
zbTRIM25-Myc-NotI-R	ATAAGAATGCGGCCGCCAAACAGAGCAAG TCTTT	pCMV-Myc-zbTRIM25
zbTRIM25-Flag-XhoI-F	CCGCTCGAGGTGCGGAACAAATGTCTCTG	pCMV-Flag-zbTRIM25
zbTRIM25-Flag-NotI-R	ATAAGAATGCGGCCGCCAAACAGAGCAAG TCTTT	pCMV-Flag-zbTRIM25
zbRIG-I-Myc-EcoRI-F	GGAATTCGGTACGAGCTGGAGAAGGA	pCMV-Myc-zbRIG-I
zbRIG-I-Myc-KpnI-R	GGGGTACCTCAGTTGACCAGCGCCCA	pCMV-Myc-zbRIG-I
zbRIG-I-Flag-EcoRI-F	GGAATTCGGTACGAGCTGGAGAAGGA	pCMV-Flag-zbRIG-I
zbRIG-I-Flag-KpnI-R	GGGGTACCTCAGTTGACCAGCGCCCA	pCMV-Flag-zbRIG-I
zbRIG-I-2CARD-EcoRI-F	GGAATTCGGTACGAGCTGGAGAAGGA	pEGFP-zbRIG-I-2CARD
zbRIG-I-2CARD-KpnI-R	GGGGTACCTCAATCTGTCTCCAGCAGG	pEGFP-zbRIG-I-2CARD
zbRIG-I-△2CARD-EcoRI-F	GGAATTCGGGGAGCAGGAAAGTCAGTGC	pEGFP-zbRIG-I-△2CARD
zbRIG-I-△2CARD-KpnI-R	GGGGTACCTCAGTTGACCAGCGCCCA	pEGFP-zbRIG-I-△2CARD
zbRIG-I-RD-EcoRI-F	GGAATTCTATGAGCTTCAAGCTCCTCTGCG	pEGFP-zbRIG-I-RD
zbRIG-I-RD-KpnI-R	GGGGTACCCTCCGTCATGTCAAACTCTC	pEGFP-zbRIG-I-RD
zbRIG-I-△(2CARDs+RD)-EcoRI-F	GGAATTCTATGGGAGCAGGAAAGTCA	pEGFP-zbRIG-I-△(2CARDs+RD)
zbRIG-I-△(2CARDs+RD)-KpnI-R	GGGGTACCGTCCTGTGTTTTGGGTTT	pEGFP-zbRIG-I-△(2CARDs+RD)
zbTRIM25-SPRY-XhoI-F	CCGCTCGAGGTTTTGATGTCAGAACGGCTC A	pCMV-Flag-zbTRIM25-SPRY

zbTRIM25-SPRY-NotI-R	ATAAGAATGCGGCCGCTCAGCACAGAGAG ACAGAGGAAC	pCMV-Flag-zbTRIM25-SPRY
zbTRIM25-△SPRY-EcoRI-F	GGAATTCTATGTTAACATGCAGCATCTGTTT	pCMV-Flag-zbTRIM25-△SPRY
zbTRIM25-△SPRY-NotI-R	ATAAGAATGCGGCCGCTCAATGGAGAATAG TGCCGTATT	pCMV-Flag-zbTRIM25-△SPRY
zbRIG-I-His-EcoRI-F	CGGAATTCATGTACGAGCTGGAGAAGGAG	pET32a-zbRIG-I
zbRIG-I-His-SalI-R	ACGCGTCGACGGTT GAC CAG CGC CCA TGT CTC	pET32a-zbRIG-I
zbTRIM25-F	GACGACCCTGCGAACTATCC	QPCR of zbTRIM25
zbTRIM25-R	CCAGCAAGATGCCAACACG	QPCR of zbTRIM25
zbISG15-F	AAAACTGCTTGGCGGTGA	QPCR of ISG15
zbISG15-R	GGAGTTCAGCCCGTAGCTGC	QPCR of ISG15
zbIFN 1-F	GCTCTGCGTCTACTTGCGAA	QPCR of IFN 1
zbIFN 1-R	GCGGCTTGGAAATGGTGT	QPCR of IFN 1
zbIRF3-F	CAAAACCGCTGTTCGTGCC	QPCR of IRF3
zbIRF3-R	TCGTCGCTGTTGGAGTCCTG	QPCR of IRF3
zbMAVS-F	CAGGCGAGAGAACTGGATTG	QPCR of MAVS
zbMAVS-R	CAGGTGGCATTAGCAGAGGAA	QPCR of MAVS
zbRIG-I-F	CGCCAGTCCTGATAAACCCA	QPCR of RIG-1
zbRIG-I-R	GCAGCGGCAGATCCTGTATG	QPCR of RIG-I
zbTRAF3-F	ATGTCCGCAGGGCGTAAT	QPCR of TRAF3
zbTRAF3-R	ACACTCAGTCTGGCGTGGGT	QPCR of TRAF3
